# NAD pool as an antitumor target against cancer stem cells in head and neck cancer

**DOI:** 10.1186/s13046-023-02631-2

**Published:** 2023-03-03

**Authors:** Lola E. Navas, Elena Blanco-Alcaina, Elisa Suarez-Martinez, Eva M. Verdugo-Sivianes, Asuncion Espinosa-Sanchez, Laura Sanchez-Diaz, Eduardo Dominguez-Medina, Ceres Fernandez-Rozadilla, Angel Carracedo, Lindsay E. Wu, Amancio Carnero

**Affiliations:** 1 Instituto de Biomedicina de Sevilla (IBIS)/HUVR/CSIC, Hospital Universitario Virgen del Rocío, Ed. IBIS, Consejo Superior de Investigaciones Científicas, Universidad de Sevilla, Avda. Manuel Siurot S/N, 41013 Seville, Spain; 2 CIBER de Cancer (CIBERONC), Instituto de Salud Carlos III, Madrid, Spain; 3 BioFarma-USEF Research Group, Center for Research in Molecular Medicine and Chronic Diseases (CIMUS), University of Santiago de Compostela, Santiago de Compostela, Spain; 4 Grupo de Medicina Xenómica (USC), Fundación Pública Galega de Medicina Xenómica, Instituto de Investigación Sanitaria de Santiago (IDIS), Santiago de Compostela, Spain; 5 CIBER de Enfermedades Raras (CIBERER), Instituto de Salud Carlos III, Madrid, Spain; 6 School of Medical Sciences, UNSW Sydney, Sydney, NSW Australia

## Abstract

Head and neck squamous cell carcinoma (HNSCC) is a heterogeneous group of tumors that affect different anatomical locations. Despite this heterogeneity, HNSCC treatment depends on the anatomical location, TNM stage and resectability of the tumor. Classical chemotherapy is based on platinum-derived drugs (cisplatin, carboplatin and oxaliplatin), taxanes (docetaxel, paclitaxel) and 5-fluorouracil^1^. Despite advances in HNSCC treatment, the rate of tumor recurrence and patient mortality remain high. Therefore, the search for new prognostic identifiers and treatments targeting therapy-resistant tumor cells is vital. Our work demonstrates that there are different subgroups with high phenotypic plasticity within the CSC population in HNSCC. CD10, CD184, and CD166 may identify some of these CSC subpopulations with NAMPT as a common metabolic gene for the resilient cells of these subpopulations. We observed that NAMPT reduction causes a decrease in tumorigenic and stemness properties, migration capacity and CSC phenotype through NAD pool depletion. However, NAMPT-inhibited cells can acquire resistance by activating the NAPRT enzyme of the Preiss-Handler pathway. We observed that coadministration of the NAMPT inhibitor with the NAPRT inhibitor cooperated inhibiting tumor growth. The use of an NAPRT inhibitor as an adjuvant improved NAMPT inhibitor efficacy and reduced the dose and toxicity of these inhibitors. Therefore, it seems that the reduction in the NAD pool could have efficacy in tumor therapy. This was confirmed by in vitro assays supplying the cells with products of inhibited enzymes (NA, NMN or NAD) and restoring their tumorigenic and stemness properties. In conclusion, the coinhibition of NAMPT and NAPRT improved the efficacy of antitumor treatment, indicating that the reduction in the NAD pool is important to prevent tumor growth.

**Supplementary Information:**

The online version contains supplementary material available at 10.1186/s13046-023-02631-2.

## Introduction

Head and neck squamous cell carcinoma (HNSCC) is the sixth most common cancer worldwide. There were almost one million new cases in 2020. This cancer is a group of tumors that affect different anatomical locations, including the oral cavity, tongue, pharynx, larynx and salivary glands. Ninety percent of these tumors originate in squamous cells^1−3^. Despite this heterogeneity, HNSCC can be classified into two large groups depending on their origin: human papillomavirus (HPV)-positive tumors and HPV-negative tumors, which are related to chronic alcohol and tobacco consumption^4,5^. HNSCC treatment depends on the anatomical location, TNM stage and resectability of the tumor. In general, early-stage tumors (T1 and T2) are usually resolved with localized surgery or radiotherapy, while more advanced-stage tumors (T3 and T4) require combinations of surgery, radiation and chemotherapy. Classical chemotherapy is based on platinum-derived drugs (cisplatin, carboplatin and oxaliplatin), taxanes (docetaxel and paclitaxel) and 5-fluorouracil^1^. An alternative to this traditional therapy is the use of specific inhibitors against EGFR, mTOR and IGF1R^6−9^. Despite advances in HNSCC treatment, the rate of tumor recurrence and the patient mortality rate remain high. Therefore, the search for new prognostic identifiers and treatments targeting therapy-resistant tumor cells is vital.

According to the cancer stem cell (CSC) model, tumors are highly heterogeneous entities that are hierarchically organized into different subgroups of tumor cells. At the top of this hierarchy is a small subpopulation of cells called tumor-initiating cells (TICs) or CSCs. Similar to normal stem cells, CSCs have the capacity to self-renew and differentiate into progenitor cells but in an uncontrolled manner. CSCs are responsible for tumor initiation, progression, metastasis and resistance to chemotherapy and radiotherapy^10−13^. Therefore, its elimination is the landmark for cancer therapy. CSCs can originate from normal stem cells or from differentiated tumor cells that acquire stem-like properties through a dedifferentiation mechanism. This dedifferentiation process is similar to iPSC (induced pluripotent stem cell) reprogramming^10,14^. The interconversion capacity between tumor cell/CSC and somatic cell/iPSC phenotypes is known as cell plasticity. The cellular microenvironment and some signaling pathways activate and regulate this cellular plasticity^11,13,15^. CSCs express specific antigens on their surface, which depend mainly on the type of cancer. The known CSC markers include CD44 + CD24- in breast cancer, CD44 + and CD133 + in colon and gastric cancer, CD34 + CD38- in leukemia, and CD133 + in glioblastoma and sarcoma^16,17^. However, within the CSC population, there are different subgroups even in the same tumor, which contributes to the high degree of heterogeneity of these cells and makes correctly identifying CSCs by their specific markers difficult^11,13,15^. Therefore, to understand the targets belonging to these cells, their inhibition, recovery, resistance, minimal residual pools, and so on must be studied.

One of the hallmarks of cancer is metabolic reprogramming to support a high proliferation rate and biomass production, which are required for tumor development and survival^10,18^. Thus, cancer cells rely on glycolysis and some glycolysis-dependent pathways, including the pentose phosphate pathway (PPP), glutaminolysis and serine and fatty acid synthesis, which allow the cells to produce macromolecules and counteract the oxidative stress caused by accelerated proliferation^19−21^. All of these upregulated pathways require the essential metabolite nicotinamide adenine dinucleotide (NAD^+^), which is implicated in numerous redox and nonredox processes, including DNA repair, cell signaling, posttranslational modifications, inflammatory responses, senescence and apoptosis^10,22–24^. Mammalian cells must synthesize NAD^+^ from different dietary precursors: tryptophan (Trp) through the de novo pathway, nicotinic acid (NA) through the Preiss-Handler pathway, and nicotinic acid riboside (NAR) or nicotinamide riboside (NR) through the nucleoside pathway^22,25^. However, cells rely on the salvage pathway as the main source of NAD^+^. SIRTs, PARPs and other enzymes consume NAD^+^ as ADP donors, releasing nicotinamide (NAM) as a catabolic product. Cells recycle NAM to reconstitute the NAD molecule again in the salvage pathway. Nicotinamide phosphoribosyltransferase (NAMPT) catalyzes the first limiting reaction, converting NAM into NMN (nicotinamide mononucleotide)^26–28^. To satisfy glycolytic and nonglycolytic cellular demands, cancer cells must increase the NAD^+^ pool, enhancing its biosynthesis.^22,29^ Indeed, high NAD^+^/NADH and NADP^+^/NADPH ratios have been found in cancer cells, suggesting that NAD plays an important role in tumor metabolism^10,30^.

As NAMPT is the key enzyme in NAD regulation, it has been proposed as an oncogene and therapeutic target in several types of cancer, including hematologic malignancies^31,32^, colon cancer^33^, prostate cancer^34^, breast cancer^35^, thyroid cancer^36^, gastric cancer^37^, and glioblastoma^27,38^. However, the therapeutic target role of NAMPT in HNSCC has not yet been evaluated. NAMPT enriches the tumor CSC subpopulation mainly through the salvage pathway, allowing an adequate supply of NAD^+^.^10,26^ NAMPT and NAD + are involved in pluripotency and dedifferentiation to CSC processes and in epithelial-mesenchymal transition (EMT), in which epithelial cells lose their cell polarity and adhesion to become mesenchymal cells with migratory and invasive properties.^33,38^

In this work, we described the plasticity of different CSC subpopulations in HNSCC tumor cell lines, with NAMPT being a common marker increased in all of them. HNSCC tumor cells with NAMPT downregulation showed greatly reduced tumorigenic capabilities in vitro and in vivo. NAMPT inhibition reduces tumor growth in vivo and combines with cisplatin and docetaxel in antitumor effects. The resistance to NAMPT downregulation becomes activated with time due in general to an increase in NAPRT or in the remaining NAMPT levels replenishing the NAD + pools. Further inhibition of both enzymes depleting NAD confirmed the antitumor effects of inhibiting NAD synthesis routes as an effective antitumor therapy for HNSCC.

## Results

### CSC pools in HNSCC cell lines

HNSCC is characterized by a high degree of heterogeneity, which greatly complicates the identification of CSC subpopulations.^39^ Therefore, we decided to validate some superficial markers proposed in the literature for HNSCC in two human tumor cell lines with different phenotypes and tissue origins, examples of HNSCC heterogeneity: RPMI-2650 – from nose carcinoma – and Detroit-562 – from larynx carcinoma. We analyzed the percentage of cells positive for CD10, CD184, CD19, CD133, CD166 and CD44 by FACS (Supplementary Table 1). All cell populations were CD44 + in both cell lines (99,68 ± 0,20 and 99,76 ± 0,17) and CD166 in Detroit-562 cells (99,63 ± 0,23), but not all cells could regenerate the culture. Therefore, we decided to validate only the CD10 + , CD184 + , CD19 + , and CD133 + pools in both cell lines and CD166 + only in RPMI-2650.

**Fig. 1:**
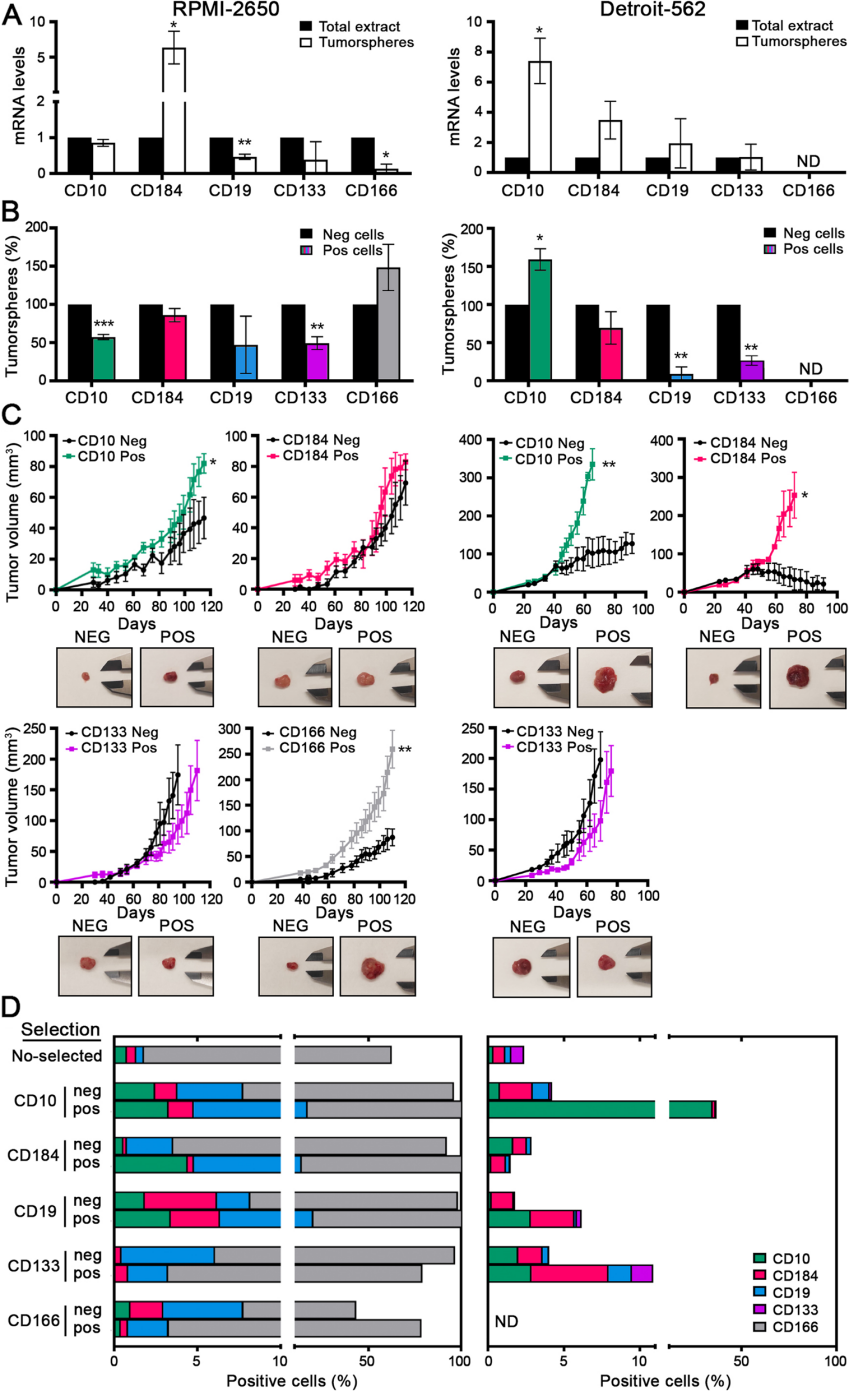
Stem properties of CD10, CD184, CD19, CD133 and CD166 subpopulations in HNSCC cell lines. A) Measurement of *CD10*, *CD184*, *CD19*, *CD133* and *CD166* expression levels by RT‒qPCR in tumorspheres (TO) and total extracts (TE) of RPMI-2650 and Detroit-562 cells. B) Tumorsphere assay of positive and negative subpopulations isolated by cytometer. C) Tumor growth in vivo in xenografts (*N* = 6) from positive and negative subpopulations isolated by a cytometer. Representative images of tumor size are shown. The mean ± standard deviation is presented. Statistical analysis was performed with Student’s t test (*p < 0.05; **p < 0.01; ***p < 0.001). ND: not determined. D) Cellular plasticity of the different subpopulations in HNSCC. Analysis of the positive cells for CD10, CD184, CD19, CD133 and CD166 markers by FACS of the isolated positive and negative subpopulations after being separated by cytometry and cultured for 1 or 2 weeks depending on the cell line. Non selected cells were used as a control. ND: not determined

First, we performed a serum-free 3D suspension “tumorsphere” culture assay. We observed that the RPMI-2650 tumorspheres expressed high mRNA levels of *CD184*, whereas the Detroit-562 tumorspheres expressed high levels of *CD10*, *CD184* and *CD19* compared with the total cellular population (Fig. 1A). The positive and negative subpopulations of each marker were isolated by cytometry and plated for tumorsphere assays. The CD166-positive subpopulation of RPMI-2650 cells and the CD10-positive subpopulation of Detroit-562 cells were able to form a slightly higher number of tumorspheres than the negative subpopulations (Fig. 1B). Then, to validate the in vivo effect of these markers, the same low number of positive and negative cells were injected as xenografts in nude mice. We observed that the tumors formed from CD10 + , CD184 + or CD166 + cells grew faster and more aggressively than the tumors formed from negative cells, especially in the case of the Detroit-562 cell line (Fig. 1C). Although we found great variability among pools and cell lines in the in vitro results, the in vivo data suggest that CD10, CD184 and CD166 could be markers related to cancer stem-like cells in our cell lines. The data also suggest that the different markers identify independent pools.

Since our results indicate that there is not a single CSC population in our individual HNSCC cell lines, we wondered if the different subpopulations studied were completely independent of whether they had any connection. For that purpose, the positive and negative cells were isolated by cytometry according to their marker expression. Then, they were cultured for 1 or 2 weeks (depending on the growth ratio of each cell line) under standard 10% serum conditions and analyzed again by FACS. Although with some variability depending on the cell line, all positive populations were able to regenerate the different positive subpopulations of the other markers, and consequently, they restored the heterogeneity of the original culture. The negative populations were also able to reconstitute the culture because they contained positive cells of the other markers and, probably, some markers not identified in this work. These data suggest a high cellular plasticity between the different subpopulations **(Fig. ****1****D)**, which makes the characterization of the therapeutic cell target pools difficult.

### NAMPT as a CSC marker in HNSCC cell lines

**Fig. 2:**
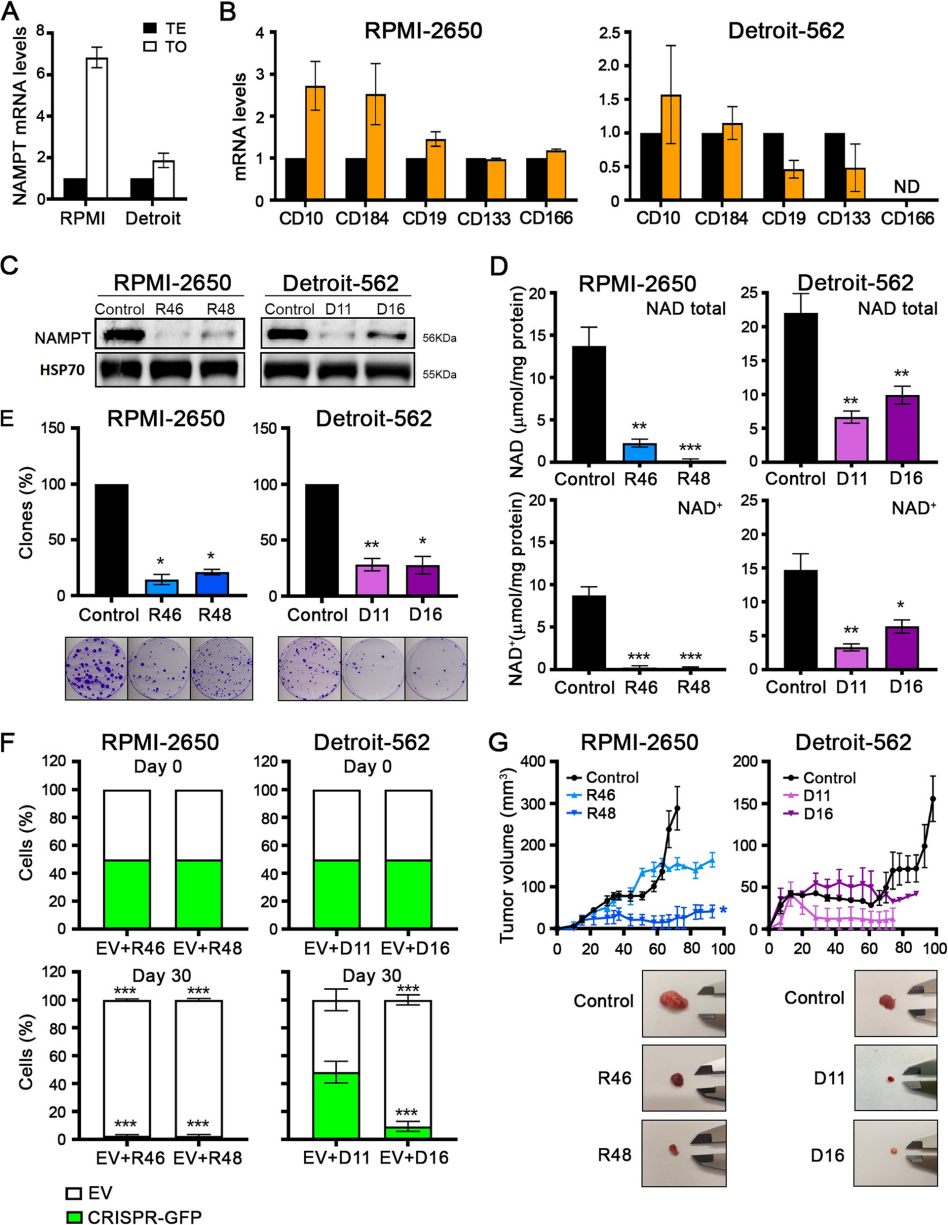
The reduction in NAMPT expression decreases tumorigenesis in HNSCC cell lines. **A** Measurement of *NAMPT* expression levels in tumorspheres and total extract by RT‒qPCR. **B** Measurement of *CD10*, *CD184*, *CD19*, *CD133* and *CD166* expression levels in NAMPT-overexpressing and EV cells by RT‒qPCR. Control cells (parental + vector only in black; overexpressing NAMPT in orange). Further validation of NAMPT overexpression is shown in Supplementary Fig. 1. **C**) WB showing reduced expression of NAMPT in NAMPT CRISPR clones, which were 2 different clones for each cell line. **D** Reduced levels of NAD + and NAD-total pools in NAMPT CRISPR clones with respect to parental cultures. **E** Reduced clonability in NAMPT CRISPR clones with respect to parental cultures. Representative images of the clones are shown. **F** Competition assay of control and NAMPT-CRISPR-GFP cells. Equal numbers of cells were seeded in the same plate, and 30 days later the GFP-cell percentage was quantified by FACS. NAMPT CRISPR clones have a lower ability to compete against parental cultures (with higher NAMPT expression) in normal culture media. Only clone D11 showed a competition capacity similar to that of the parental cells. **G** Tumor growth and tumor size of tumors from parental or NAMPT CRISPR clones. Tumor growth in vivo in xenografts (*N* = 4) from NAMPT CRISPRs and control cells. Representative images of tumor size are shown. The mean ± standard deviation is presented. Statistical analysis was performed with Student’s t test (*p < 0.05; **p < 0.01; ***p < 0.001)

As NAMPT and NAD^+^ are involved in the dedifferentiation to CSCs in many tumors^10, 26, 33^, we also validated NAMPT as a possible marker in HNSCC. We found an increase in *NAMPT* mRNA levels in tumorspheres compared with the total extract in both cell lines (Fig. 2A), indicating that 3D cultures enriched in CSCs were also enriched in the expression of NAMPT. As a counterpart, at the transcriptional level, we also found higher levels of *CD10, CD184* and *CD166* mRNA in NAMPT-overexpressing cells by cDNA ectopic transfection (Fig. 2B and Supplementary Fig. 1).

Additionally, we wondered about the clinical relevance of the marked studies – CD10, CD184, CD19, CD133, CD166 and NAMPT. To answer this question, we analyzed the overall survival of HNSCC patients in The Cancer Genome Atlas (TCGA) database. We found that high levels of NAMPT were correlated with poor patient survival. NAMPT seems to be an indicator of poor prognosis in HNSCC, as occurs in other types of tumors (Supplementary Fig. 2). These data also indicate that NAMPT could be a more suitable marker for clinical prognosis.

### Effect of NAMPT CRISPRs on the tumorigenic properties of HNSCC cell lines

Therefore, we decided to study the effect of decreased NAMPT expression on the tumorigenic properties of our HNSCC cell lines. First, we generated NAMPT knockdown models using the CRISPR/Cas9 system. The clones R46 and R48 in RPMI-2650 and D11 and D16 in Detroit-562 were validated by western blotting to measure the protein levels of NAMPT (Fig. 2C). We also validated the reduction in the enzymatic activity of the proposed NAMPT CRISPRs by quantifying the NAD^+^ and NAD total pools (Fig. 2D).

Once the CRISPRs were validated, we performed functional assays to validate the role of this reduction in tumorigenic properties. In the clonability assay, NAMPT CRISPR cells formed a smaller number of clones than control cells (Fig. 2E). Then, we evaluated the competition capacity of cells under the same conditions. We transfected NAMPT CRISPRs with the EYFP-Mito plasmid, which expresses GFP (green fluorescent protein). Then, we seeded NAMPT CRISPR-transfected cells and EV cells in the same dish. After 30 days, we observed a significant reduction in the percentage of GFP-positive cells, except in the coculture of EV-CRISPR D11 of the Detroit-562 cell line (Fig. 2F). Next, cells were injected as xenografts in nude mice. NAMPT CRISPRs formed smaller tumors that grew slower than tumors formed from control cells (Fig. 2G). We can conclude that the reduction in NAMPT expression decreases some tumoral properties in our HNSCC cell lines.

### Effect of NAMPT CRISPRs on the stemness properties of HNSCC cell lines

**Fig. 3:**
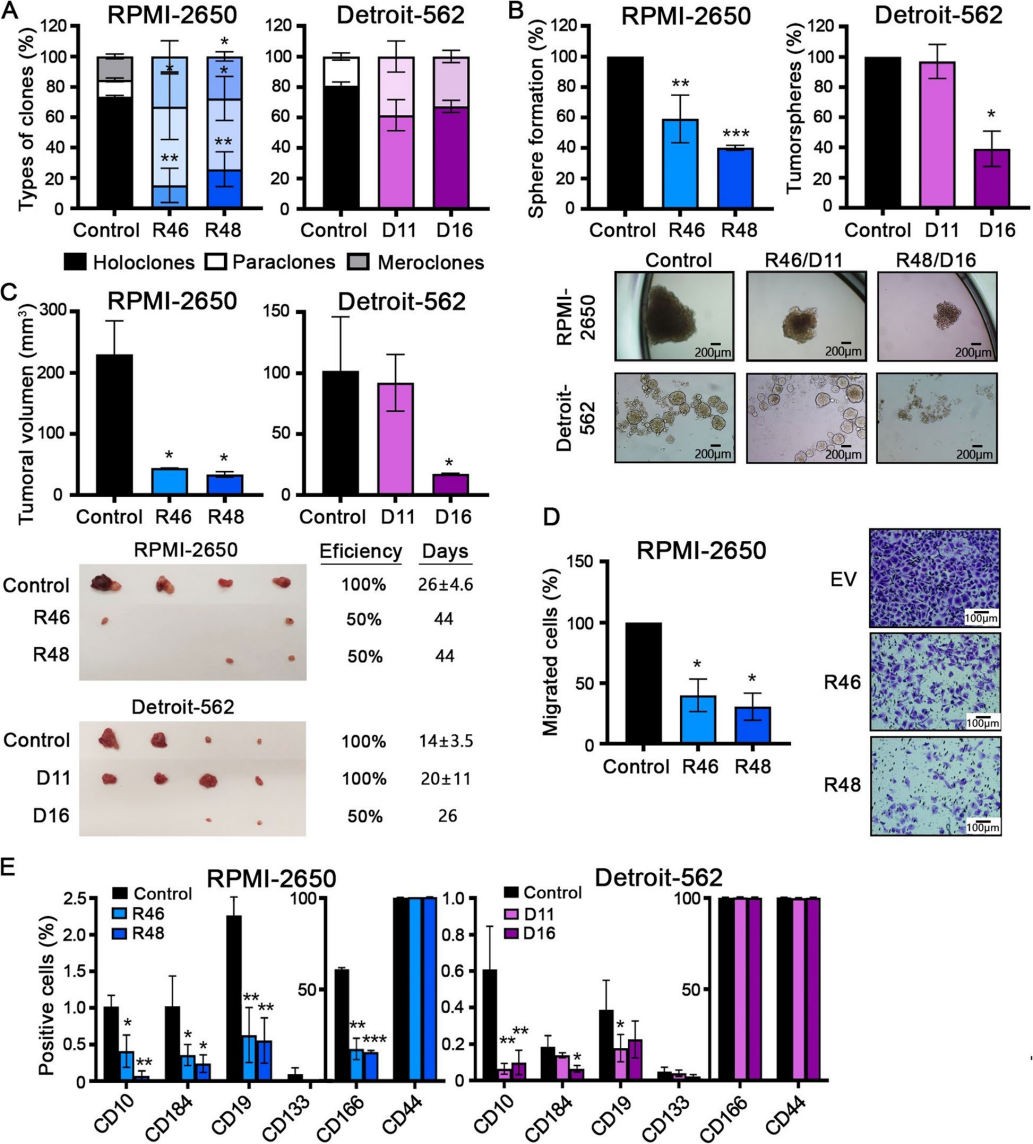
The reduction in NAMPT expression decreases stemness in HNSCC cell lines. **A** Analysis of the clone phenotypes (holoclones, meroclones and paraclones) formed by NAMPT CRISPRs and control cells in RPMI-2650 and Detroit-562 cell lines. In each solid barr, bottom part are holoclones, medium part are paraclones and upper part are meroclones. In Detroit, no meroclones were observed. **B** Tumorsphere assay from single-cell sorting in RPMI-2650 or the whole cell population in Detroit-562. Representative images of the tumorspheres formed are shown. **C** Tumorigenicity of tumorspheres in vivo. Tumorspheres from NAMPT CRISPRs and control cells were injected into xenografts (*N* = 4). Images of the tumors, the efficiency of tumor formation and the time required to appear are presented. **D** Analysis of cell migration in NAMPT CRISPRs and control cells by Boyden Chamber assay. Representative microscopic images are shown (20X). **E** Analysis of positive and negative CD10, CD184, CD19, CD133, CD166 and CD44 subpopulations in NAMPT CRISPRs and control cells by FACS. The mean ± standard deviation is represented in all cases. Statistical analysis was performed with Student’s t test (*p < 0.05; **p < 0.01; ***p < 0.001)

Previously, we observed that NAMPT is a marker related to the CSC population; therefore, we wanted to study how reduced NAMPT expression can affect stemness properties. In the clonogenic assay, we found that NAMPT CRISPRs formed a lower percentage of holoclones and a higher percentage of paraclones, which are enriched in differentiated tumor cells, compared with control cells, although these differences were not significant in the Detroit-562 cell line (Fig. 3A). We also observed that NAMPT CRISPRs cells formed a smaller and lower number of tumorspheres from single-cell sorting in RPMI-2650 or the whole cell population in Detroit-562. However, CRISPR D11 of Detroit-562 cells showed the same tumorsphere-formation capacity as control cells (Fig. 3B). Then, we injected the tumorspheres formed as xenografts in nude mice. The efficiency of tumor formation from NAMPT CRISPRs tumorspheres was only 50%; these tumors were smaller and required more days to form, except for the tumors of CRISPR D11 tumorspheres, which had 100% efficiency and were similar to the control tumors (Fig. 3C). We also studied the effect of reduced NAMPT expression on cell migration capacity. The NAMPT CRISPRs of RPMI-2650 cells showed a lower percentage of migrated cells in the Boyden chamber assay (Fig. 3D). Next, we measured the effect of the reduction in NAMPT in the different CSC pools in both cell lines (Fig. 3E). We found that in all cases, except CD44 + in both cell lines or CD166 + only in Detroit, reduction of NAMPT led to a reduction in the percentage of CSCs in the pools analyzed. These data confirm the relevance of NAMPT as a target in CSCs in HNSCC cell lines (Fig. 3E).

On the other hand, NGS (next-generation sequencing) of the differentially expressed genes between NAMPT overexpression and CRISPRs was performed. The GO terms of the genes obtained from the sequencing indicated that NAMPT is mainly related to metabolic processes; NAMPT can bind to proteins, poly(A)RNA, nucleotides, ribonucleotides and ATP; and NAMPT can alter the transcription of some cellular components (e.g., stress fiber, centrosome, intercellular bridge and nucleus) (Supplementary Fig. 3). In addition, we performed a transcriptomic analysis to identify genes common to CD10 + , CD184 + , CD19 + and NAMPT + in our HNSCC cell lines by quantitative NGS sequencing. Differential genes between the positive and negative subpopulations and NAMPT-overexpressing and NAMPT CRISPRs cells were analyzed. We obtained 3 genes common to all of the pools: 13 genes common to NAMPT, CD10 and CD184; 36 genes common to NAMPT and CD10; 63 genes common to NAMPT and CD184; and genes common 18 to NAMPT and CD19. Most of the resulting genes are related to tumorigenesis, cell proliferation, metastasis and treatment resistance (Supplementary Fig. 4 and Supplementary Table 2). In conclusion, all of these data agree with the results obtained previously, suggesting that NAMPT could be a common CSC marker to CD10, CD184, CD19, CD133 and, especially, CD166.

### NAMPT as a therapeutic target in HNSCC cell lines

**Fig. 4:**
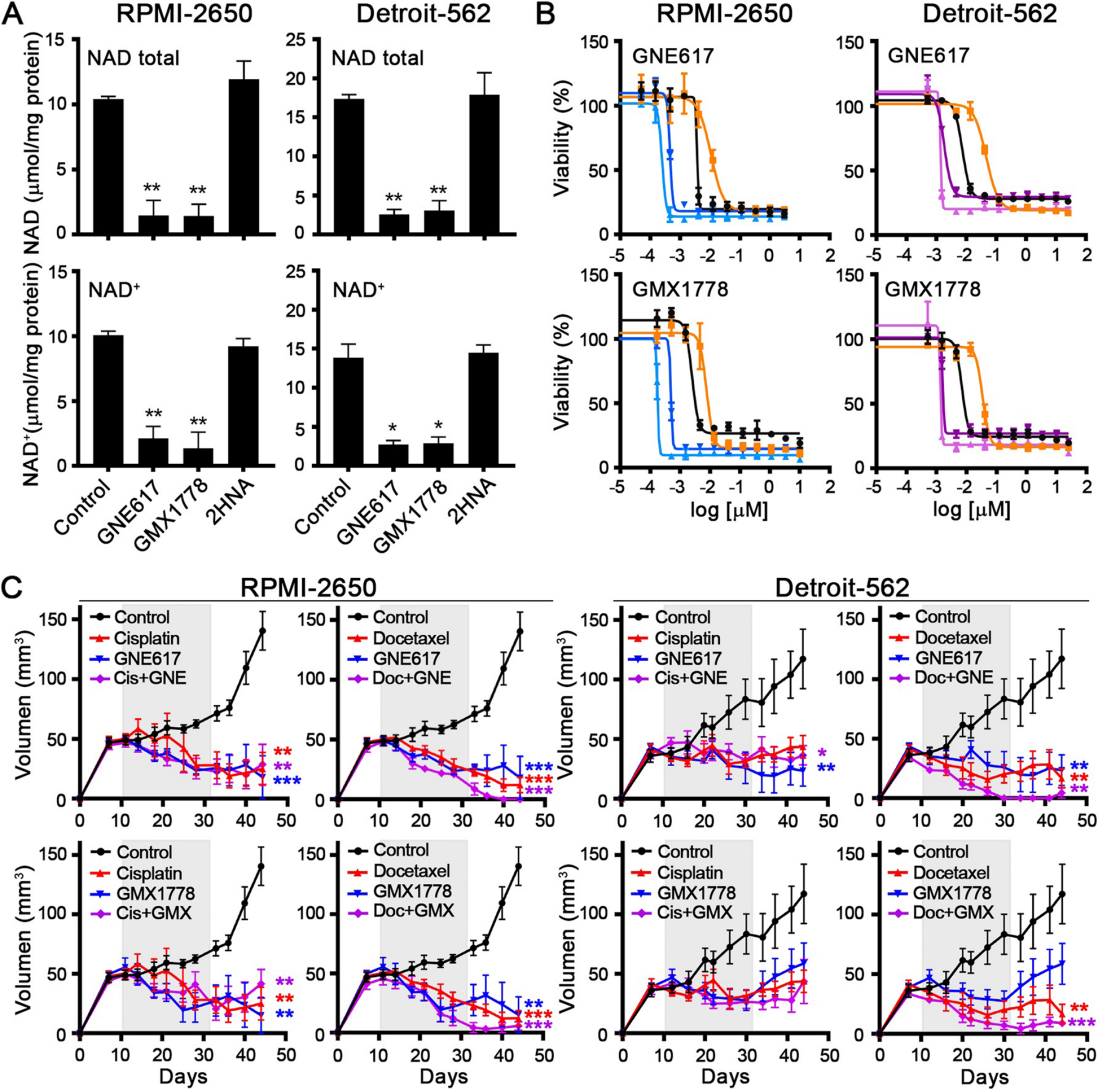
NAMPT as a therapeutic target in HNSCC cell lines. **A** Total NAD and NAD.^+^ quantification after 24 h of treatment with the NAMPT inhibitors GNE617 (10 nM) and GMX1778 (10 nM) and the NAPRT inhibitor 2HNA (1 mM). **B** IC50 of GNE617 and GMX1778 in NAMPT-overexpressing and NAMPT CRISPR cells. In black, parental cells. In orange curve of cells overexpressing NAMPR by ectopic transfection. In blue, CRISP clones of RPMI cells downregulating NAMPT. In Purple, CRISP clones of DETROIT cells downregulating NAMPT. **C** Treatment with NAMPT inhibitors (in blue), traditional drugs (in red) in monotherapy and in combination (in purple) in xenografts from RPMI-2650 and Detroit-562 cells. In each cohort, *N* = 6. The following drug doses were used: 30 mg/kg of GNE617 v.o. 5 consecutive days followed by 5 days off, 200 mg/kg GMX1778 v.o. once a week, 2 mg/kg of cisplatin i.p. 4 times a week and 15 mg/kg docetaxel i.p. twice a week. All treatments lasted 3 weeks (gray band), and the tumor measurement was continued for two more weeks. The mean ± standard deviation is presented. Statistical analysis was performed with Student’s t test (*p < 0.05; **p < 0.01; ***p < 0.001)

Given the oncogenic role of NAMPT, several specific inhibitors have been developed in recent years. In this work, we validated the inhibitors GNE617 and GMX1778, two oral compounds that competitively inhibit NAMPT, as most compounds are currently being tested. The GNE617 and GMX1778 treatment produced a total depletion of the NAD pool in parental cells (Fig. 4A). In contrast, treatment with 2-hydroxynicotinic acid (2HNA) alone, an inhibitor of NAPRT, did not produce any significant change in NAD levels (Fig. 4A). These data confirm that cells rely on NAMPT activity, rather than NAPRT, for NAD biosynthesis. Then, we evaluated the cytotoxic effect of GNE617 and GMX1778 inhibitors by IC50 assay, which determined the drug concentration required to inhibit 50% of cells. In both cell lines, NAMPT-overexpressing cells were more resistant to NAMPT inhibitors, whereas CRISPR cells were more sensitive to drug action (Fig. 4B, supplementary Table 3). Then, we studied the effect of GNE617 and GMX1778 in vivo in monotherapy and in combination with cisplatin or docetaxel, two classical chemotherapeutic agents used to treat patients with HNSCC. We generated xenografts from RPMI-2650 and Detroit-562 cells. When the tumors were well established, the mice were randomized into different groups and treated for three weeks. We observed a significant tumor reduction in all of the treatments; hence, GNE617 and GMX1778 were as effective as conventional drugs used in monotherapy. Moreover, the GNE617 and GMX1778 treatments enhanced the antitumor effect of docetaxel until the complete elimination of tumors in mice (Fig. 4C). These results suggest that the NAMPT inhibitor could be a candidate for monotherapy or an adjuvant to current HNSCC therapy; however, more studies are needed to confirm this hypothesis.

### Mechanism of NAMPT CRISPR resistance

**Fig. 5:**
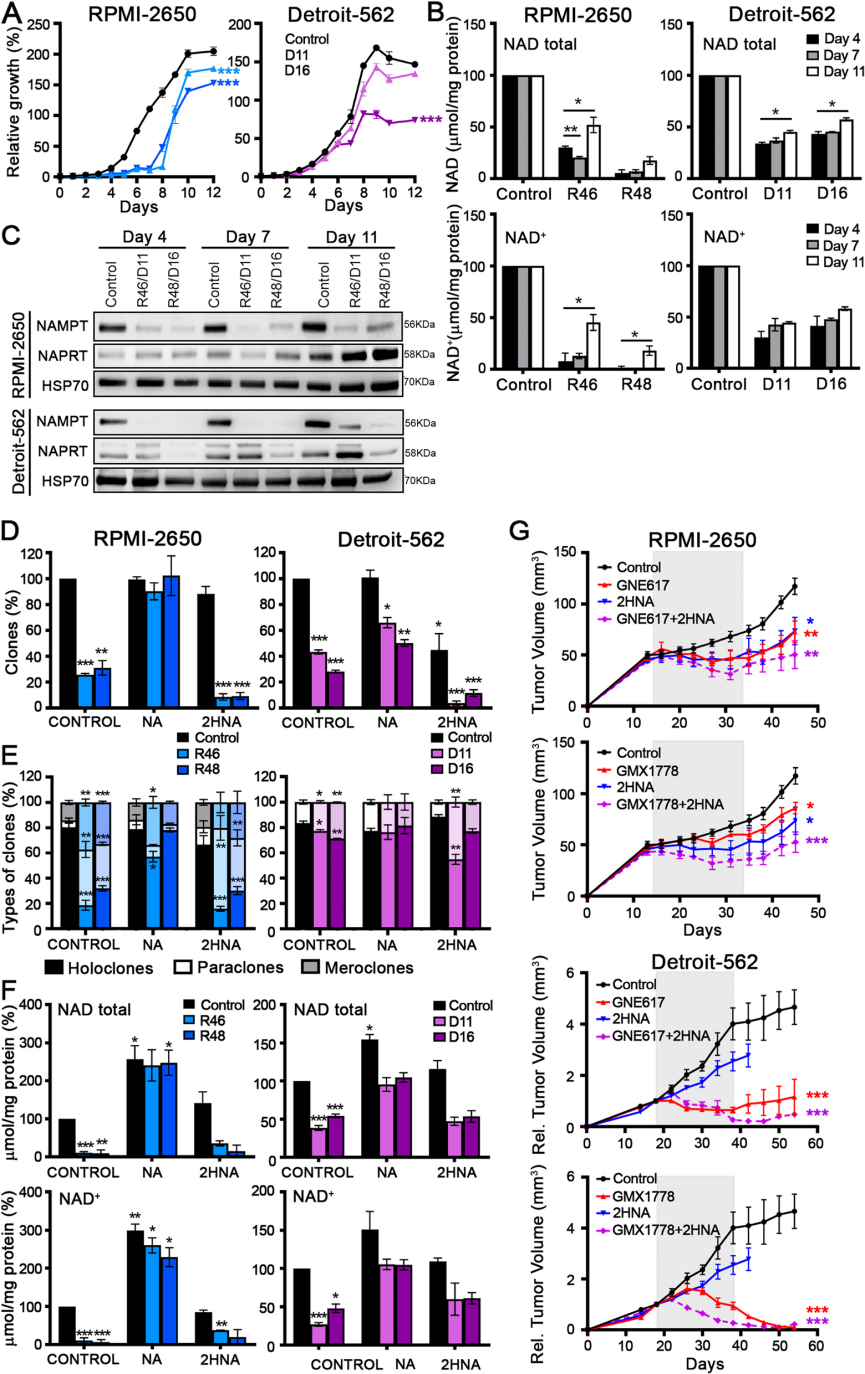
Proliferation rate recovery of NAMPT CRISPRs during the growth curve in HNSCC cell lines. **A** Growth curves of NAMPT CRISPRs and control (empty vector only, EV) cells of RPMI-2650 and Detroit-562 cell lines. **B** Total NAD and NAD + quantification of NAMPT CRISPRs and (empty vector only, EV) cells at three different points on the growth curve: Day 4, Day 7 and Day 11. The mean ± standard deviation is presented. Statistical analysis was performed with Student’s t test (*p < 0.05; **p < 0.01; ***p < 0.001). **C** Analysis of NAMPT and NAPRT protein levels by Western blotting at Day 4, Day 7 and Day 11. **D**, **E** & **F** NAPRT as a resistance mechanism in NAMPT CRISPRs in HNSCC cell lines. **D** Clonogenic assay, **E)** clonal phenotype analysis. Analysis of the clone phenotypes (holoclones, meroclones and paraclones) formed by NAMPT CRISPRs and control cells in RPMI-2650 and Detroit-562 cell lines. In each solid bar, bottom part are holoclones, medium part are paraclones and upper part are meroclones. In Detroit, no meroclones were observed. **F** NAD quantification of NAMPT CRISPRs and (empty vector only, EV) cells under normal conditions (CONTROL) after nicotinic acid treatment (NA, 0.5 mM) and treatment with the NAPRT inhibitor 2-hydroxynicotinic acid (2HNA, 1 mM). 200 mg/kg 2HNA i.p. four times a week. **G** In vivo reduction of tumors by the NAMPT inhibitors GNE617 and GMX1778 in monotherapy and in combination with 2HNA (1 mM) in parental RPMI-2650 and Detroit-562 cell lines. All treatments lasted 3 weeks (gray band), and the tumor measurement was continued for two more weeks. In each cohort, *N *= 6. The mean ± standard deviation is presented. Statistical analysis was performed with Student’s t test (*p < 0.05; **p < 0.01; ***p < 0.001)

We observed that NAMPT CRISPR cells showed a recovery of some tumorigenic properties, although they seemed to have reduced NAMPT expression. In the growth curve of the RPMI-2650 cell line, NAMPT CRISPR cells grew slowly in the first days; then, the proliferation rate the cells accelerated, restoring the growth of the cell population. In Detroit-562 cells, CRISPR D11 cells grew similarly to the control cells (Fig. 5A). These data suggest that NAMPT CRISPR cells were probably reboosting the NAD^+^ pool using other alternative biosynthesis pathways. To check this point, we measured the levels of total NAD or NAD + pools at different points on the growth curve, and we identified different growth properties: on Day 4, Day 7 and Day 11. We found that the RPMI-2650 CRISPR cells increased NAD and NAD + levels at the end of the curve (Fig. 5B). To determine how these cells reboosted NAD, we first checked the protein level of NAPRT, which catalyzes the first reaction of the Preiss-Handler pathway, and then we checked that of NAMPT by Western blotting at three different points in the growth curve: on Day 4, Day 7 and Day 11. We found that the RPMI-2650 CRISPR cells increased NAPRT protein levels at the end of the curve. In contrast, the Detroit-562 CRISPRs did not change their NAPRT levels, but they increased the residual protein levels of NAMPT at the end of the curve. This increase was considerably important in the case of CRISPR D11, which also had high levels of NAPRT throughout the curve (Fig. 5C). These results could justify the observed resistance of CRISPR D11 in the growth curve and in other functional assays. Therefore, the NAMPT CRISPR cells can recover their proliferative capacity by boosting their NAD pool through two different mechanisms: the CRISPRs of the RPMI-2650 cell line enhance the NAPRT activity of the Preiss-Handler pathway, and the CRISPRs of Detroit-562 also enhance the remanent NAMPT activity of the salvage pathway.

As NAPRT may act as a resistance mechanism in NAMPT CRISPRs, we decided to study the effect of nicotinic acid (NA), its substrate, on some tumorigenic properties. When we treated the cells with NA, we observed that NAMPT CRISPRs recovered the ability to form clones and the percentage of holoclones enriched in CSCs in the RPMI-2650 cell line. However, we did not observe a great change in the Detroit-562 cell line. When we treated the cells with 2HNA, the NAPRT inhibitor, we observed a decrease in the ability to form clones in the CRISPR cells in both cell lines but not in the percentage of holoclones (Fig. 5D, 5E). In addition, treatment with NA allowed NAMPT CRISPR cells to recover NAD basal levels. However, treatment with 2HNA did not reduce the cellular NAD pools (Fig. 5F). These results suggest that NAPRT activity is not essential for NAD production; however, the NAPRT pathway could be a resistance mechanism for NAMPT expression inhibition.

Indeed, we demonstrated that 2HNA coadministration made xenograft tumors in vivo more sensitive to the action of NAMPT inhibitors (Fig. 5G). In both cell lines, the coadministration of NAMPT and NAPRT inhibitors cooperated inhibiting tumor growth (Fig. 5G). Therefore, the depletion of the NAD pool by inhibiting the two main enzymes in the NAD synthesis pathways showed efficacy as antitumor therapy in two very different cell lines, with multiple pools of CSCs with high plasticity.

## Discussion

In this work, we aimed to target CSC pools with a therapy to be effective against resistance, recidiva and metastasis. Therefore, we first aimed to study CSCs in HNSCC. HNSCC is a set of tumors that affect a great diversity of different tissues, contributing to a high degree of heterogeneity. Indeed, there is a huge controversy regarding the specific markers that identify the CSC subpopulation in HNSCC^39^. We analyzed the expression of some superficial markers by flow cytometry: CD10, CD184, CD19, CD133, CD166, and CD44. However, we discarded CD166 in the Detroit-562 cell line and CD44 in both cell lines because all of the cell populations were positive for these markers. The use of CD44 as an HNSCC CSC marker is highly discussed in the literature, and its specificity appears to depend on the original tumor tissue^6,8^. Similar to our cell lines, the mouth, hypopharynx and oropharynx tumor cell lines also show a high percentage of CD44-positive cells^40^. In several studies, CD44 cannot identify oral tumor cells because both healthy and tumor tissue equally express this marker^40−42^.

The most promising markers of this work were CD166 for RPMI-2650 and CD10 and CD184 for the Detroit-562 cell line. The use of CD166 is also highly discussed because some studies have found high CD166 expression in HNSCC cell lines^43,44^. The expression pattern of CD166 probably depends on the original tissue of the tumor, in the same way as for CD44. In our cell lines, the entire population of the Detroit-562 cell line was positive for CD166, while 60% of RPMI-2650 cells were positive for this marker. Although this percentage is too high to be related to the CSC subpopulation, the tumors in xenografts formed from CD166-positive cells grew faster and more aggressively. This finding suggests that CD166 could be a tumorigenic marker related to the CSC-like phenotype in the RPMI-2650 cell line. The Detroit-562 tumorspheres showed a higher percentage of CD10- and CD184-positive cells, and the tumors in xenografts formed by these cells grew faster and more aggressively. CD10 has been proposed as a poor prognostic indicator in patients with oral carcinoma and other HNSCC subtypes^45^. In addition, high levels of CD10 have been found on cells resistant to cisplatin, fluorouracil and radiation in several HNSCC cell lines, including Detroit-562^46^. CD10 is also involved in some signaling pathways, such as the PI3K-Akt pathway,^47,48^ which is highly activated in HNSCC^5,49^. CD184 has been described as a membrane receptor mainly expressed on CSCs, also called CXCR4. The interaction of the receptor CD184/CXCR4 with its ligand SDF-1(also called CXCL12), enhances the interaction of CSCs with the tumor microenvironment^50,51^. However, we have not found studies that evaluate, as we have done, the tumorigenic capacity of the CD184-positive subpopulation in HNSCC.

The isolated CD10, CD184, CD19, CD133 and CD166 positive subpopulations were able to regenerate the different populations of the other markers, thus regenerating the heterogeneity of the original culture. This plasticity could explain why there are several CSC subpopulations within the culture, complicating the study of CSC physiology in HNSCC.

Cancer cells enhance NAD^+^ production to satisfy glycolytic and nonglycolytic demands of the tumor^10,30^. The enzyme NAMPT catalyzes the rate-limiting reaction of the NAD^+^-salvage pathway, and it has been described as an oncogene in several types of cancer^27,31–36,38^. Thus, we studied the role of NAMPT in HNSCC tumorigenesis and stemness. The overexpression of NAMPT increased the CD10- and CD184-positive subpopulations and formed more aggressive tumors in vivo in the Detroit-562 cell line. On the other hand, the reduction in NAMPT expression causes a decrease in the proliferation rate, clone and tumorsphere formation, competition with other cells, in vivo proliferation and tumor formation in xenografts. Cells with reduced NAMPT expression showed a significant decline in the NAD pool. However, this reduction was more important in RPMI-2650 cells than in Detroit-562 cells, possibly because Detroit-562 cells had higher basal levels of NAD. Indeed, RPMI-2650 CRISPRs showed a lower tumorigenic capacity. The reduction in NAMPT expression also caused a decrease in CD10-, CD184-, CD19-, CD133- and CD166-positive subpopulations and a decline in cellular migration capacity. We observed that NAMPT downregulation reduced the tumorigenic and CSC-like properties of tumor cells in our cell lines. In addition, we found that high levels of NAMPT was correlated with worse survival in patients with HNSCC in the TCGA database. Therefore, NAMPT could be an indicator of poor prognosis in HNSCC, as occurs in gastric cancer, colon cancer and glioblastoma.^10,33,37,38^ Based on the data of this work, we propose NAMPT as a possible common marker for different CSC subpopulations in HNSCC. Therefore, we aimed to target NAMPT to explore whether its inhibition can have efficacy against HNSCC.

Treatment with GNE617 and GMX1778 NAMPT inhibitors caused an important depletion of the NAD pool similar to that produced by FK866, a noncompetitive inhibitor used as a positive control. NAMPT-overexpressing cells were more resistant and required a higher dose of these inhibitors, while NAMPT CRISPR cells were more sensitive to them. Our results coincide with those of a study in which low levels of NAMPT expression were correlated with activity of NAMPT inhibitors, including FK866 and GMX1778^52,53^. In vivo, GNE617 and GMX1778 as single treatments were just as effective as cisplatin and docetaxel, two chemotherapy drugs currently used to treat HNSCC patients. In addition, GNE617 and GMX1778 treatment enhanced the antitumor effect of docetaxel. Therefore, although NAMPT inhibitors show some toxicity in the clinic, they may be used as adjuvants to current therapies or for further development.

Despite having reduced NAMPT expression levels and reduced cellular NAD pools, the CRISPR recovered some tumorigenic properties. For example, NAMPT CRISPR cells grew slowly the first days of the growth curve, but then they were able to accelerate their rate proliferation and restore the culture in the RPMI-2650 cell line. We verified that these cells replenished the NAD pool at the end of the growth curve by two different mechanisms: the CRISPRs of RPMI-2650 increased the protein levels of NAPRT—the first enzyme of the Preiss-Handler pathway—while the CRISPRs of Detroit-562 increased the residual protein levels of NAMPT. The CRISPR D11 of Detroit-562 also had a high basal activation of NAPRT, which would justify the resistance shown in the proliferation curve and in other functional assays. Exogenous treatment with the NAPRT substrate nicotinic acid (NA) allowed the NAMPT CRISPRs of the RPMI-2650 cell line to reboost the NAD pool and, consequently, recover the proliferation, clone formation and percentage of holoclones. NA treatment also allowed the NAMPT CRISPRs of the Detroit cell line to reboost the NAD pool, but it did not increase tumorigenic properties, indicating that these cells depend more on NAMPT activity. On the other hand, the inhibition of NAPRT by 2HNA reduced the proliferation and clone-forming capacity but not the holoclones percentage, in NAMPT CRISPRs. However, the NAD pool did not decrease in response to 2HNA, suggesting that NAPRT is necessary in NAD^+^-biosynthesis only in the absence of NAMPT. The Detroit-562 line presents amplifications of the NAPRT gene (cbioportal.org/); thus, we assume that the line comes from a NAPRT-positive tumor, whose original tissue had high levels of NAPRT^54−56^. NAPRT-positive tumors have an adequate NAD + supply and show resistance to NAMPT inhibitors and alkylating agent treatment^10,55,57^. In fact, Detroit-562 cells have higher basal levels of NAD and are more resistant to NAMPT inhibitors and cisplatin, which is an alkylating agent. This would explain the observed resistance of Detroit-562 cells to the effect of the inhibitor 2HNA and the behavior of the CRISPR cells. These cells had already activated the NAPRT pathway, and they must enhance the remaining NAMPT activity. Although NAPRT inhibition does not affect the NAD pool in cells with normal levels of NAMPT, it appears that it may sensitize cells to the effect of NAMPT inhibitors.

Therefore, we tested this hypothesis in vivo. The use of an NAPRT inhibitor as an adjuvant could improve NAMPT inhibitor efficacy, mainly in NAPRT-positive tumors, and would reduce the dose and toxicity of these inhibitors^58^. In line with this, NAPRT inhibition has been reported to sensitize ovarian tumors to the inhibitory effect of NAMPT inhibitors^57^. We observed that coadministration of the NAMPT inhibitor with the NAPRT inhibitor cooperated inhibiting tumor growth. Therefore, it seems that the reduction in the NAD pool could have efficacy in tumor therapy. This can be achieved without reaching the maximal tolerated dose for each inhibitor, therefore reducing toxicity.

## Conclusions

Our work demonstrates that there are different subgroups with high phenotypic plasticity within the CSC population in HNSCC. CD10, CD184, and CD166 may identify some of these CSC subpopulations with NAMPT as a common metabolic gene for the resilient cells of these subpopulations. We observed that NAMPT reduction causes a decrease in tumorigenic and stemness properties, migration capacity and CSC phenotype through NAD pool depletion. However, NAMPT-inhibited cells can acquire resistance by activating the NAPRT enzyme of the Preiss-Handler pathway. Finally, the inhibition of both NAMPT and NAPRT improved the efficiency of the treatment, indicating that the reduction of the NAD pool is important for preventing tumor growth.

## Methods

### Cell culture

The RPMI-2650 and Detroit-562 cell lines were obtained from the ATCC commercial repository. Cells were cultured in DMEM (AQmedia: Sigma) supplemented with 10% fetal bovine serum (FBS) (Gibco), penicillin, streptomycin and fungizone (Sigma) and incubated at 37 °C in 5% CO_2_ in a humidified atmosphere. Cells were negative for mycoplasma.

### RT‒qPCR

Total RNA was isolated using the RNeasy kit (Qiagen), and cDNA was generated from 1 μg of mRNA using the High Capacity cDNA Reverse Transcription kit (Life Technologies). The PCR mixture (10 μL) contained 2μL of the cDNA diluted 1:3, 2.5μL of water, 5μL of GoTaqR Probe qPCR Master Mix (Promega) and 0.5μL of appropriate TaqMan Assay (20X) (Applied Biosystems). We used the following probes: *NAMPT* (Hs00237184_m1), CD10 (Hs00153510_m1), CD184 (Hs00607978_s1), CD19 (Hs01047413_g1), CD133 (Hs01009257_m1), CD166 (Hs.PT.56a.20098970.g) and GAPDH (Hs03929097_g1) as housekeeping genes. Relative mRNA expression is presented.

### Western blot analysis

Western blotting was performed according to standard procedures. We used the following primary antibodies: anti-NAMPT (Bethyl (A300-779A), anti-NAPRT (Sigma (SAB1400768), anti- tubulin (Sigma (T9026), and anti-HSP70 (ab45133). We used the following secondary antibodies: rabbit anti-mouse (Abcam ab97046) and goat anti-rabbit (Abcam ab97051).

### CRISPR/Cas9 to generate NAMPT knockdown

An sgRNA targeting the NAMPT sequence CTTTACATAGGACGCCAGCA (exon 10) was used to generate knockdown models. First, we infected the cells with virus containing NAMPT-sgRNA and drug selection. Then, the cells were isolated by single-cell sorting by FACS Jazz (BD Biosciences) in 96-well plates. One month later, each well that grew was amplified and validated by Western blot analysis. The selected CRISPRs were sequenced in the Genomics and Sequencing service at IBiS.

### Transfection and plasmids

Cells were transfected with TransIT-X2 (Mirus) and the following plasmids: pBabe-empty vector or pMMLV-NAMPT-Myc-His-IRES-Puro (from VectorBuilder). Transfection was performed according to the manufacturer’s instructions. After 48 h, cells were seeded with media and 1–0.5 μg/mL puromycin.

### Growth curve

To measure the proliferation capacity, 5 × 10^3^ (RPMI-2650) and 1 × 10^4^ (Detroit-562) cells were seeded in 6-well plates in triplicate. At 24 h (Day 0), the cells were fixed with 0.5% glutaraldehyde (Sigma), and every 48 h a curve point was fixed up to 12 days. Once all of the points were collected, the plates were stained with 0.5% crystal violet (Sigma). Then, the crystal violet was solubilized in 20% acetic acid (Sigma) and quantified at 595 nm absorbance as a relative measure of cell number. The values were represented referring to Day 0.

### Clonogenic assay and clonal heterogeneity analysis

To measure the ability of cells to form individual clones, 1 × 10^3^ (RPMI-2650) and 5 × 10^3^ (Detroit-562) cells were seeded in 10 cm plates in triplicate. After 10–15 days, the cells were fixed with 0.5% glutaraldehyde and stained with 0.5% crystal violet. The number of clones was counted, and the types of clones were classified according to phenotype and ability to reconstitute the culture when isolated.

### Tumorsphere assay

In the case of Detroit-562 cells, 2 × 10^4^ cells were seeded in 24-well ultra-low attachment plates (Corning) containing 1 mL of MammoCult basal medium (Stem Cell Technologies) supplemented with 10% MammoCult proliferation supplement, 4 µg/mL heparin, 0.48 µg/mL hydrocortisone, 5% penicillin:streptomycin, and 5% fungizone. After 72 h, the number of tumorspheres formed was measured using an inverted microscope (Olympus IX-71).

In the case of RPMI-2650, single cells were individually seeded through sorting with a FACS Jazz (BD Biosciences) in 96-well ultra-low attachment plates containing 1 mL of supplied MammoCult medium. After 30 days, the number of tumorspheres formed was measured.

### Competition assay

NAMPT CRISPR cells were transfected with the EYFP-Mito plasmid, which expresses a green fluorescent protein (GFP). A total of 5 × 10^5^ transfected cells and 5 × 10^5^ empty vector cells were seeded in the same dish, and the percentage of GFP-positive cells was analyzed at 24 h (Day 0) and at 30 days by FACS.

### Migration assay (Boyden chamber)

To measure the migration capacity of RPMI-2650, 4 × 10^4^ cells were resuspended in 300μL FBS-free medium and seeded in an 8 μm Boyden chamber (Transwell). The chamber was placed in a 24-well plate with FBS-medium (FBS acted as chemoattractant agent). At 24 h, the cells were fixed with 0.5% glutaraldehyde and stained with 0.5% crystal violet. The inner membrane of the chamber was cleaned to avoid nonmigrated cells being stained. Pictures were taken, and migrated cells were counted using an inverted microscope (Olympus IX-71).

### Cytotoxic assay

A total of 8 × 10^3^ RPMI-2650 cells or 16 × 10^3^ Detroit-562 cells were seeded in 96-well plates. The cells were treated 24 h later with different concentrations of the drugs: GNE617 (25–0 μM), GMX1778 (25–0 μM), cisplatin (33.3–0 μM), docetaxel (33.3–0 μM), and 2HNA (1 mM). After 96 h, the cells were stained with 0.5% crystal violet. Then, the violet crystal was solubilized in 20% acetic acid (Sigma) and quantified at 595 nm absorbance to measure the cell viability.

### Metabolism assays

NAD levels were assayed based on the NAD cycling method of Zhu and Rand, PLoS One 2012^59^. Cells were treated as indicated, media was removed, plates were washed in cold PBS and cells were scraped down in a buffer containing 50 mM Tris HCl, 0.1% Triton X-100 and 10 mM nicotinamide, to inhibit the activity of NAD-degrading enzymes. Cells were homogenised by sonication for 5 s, and samples were centrifuged at 7,000 g for 5 min at 4 degrees. Aliquots were taken for later protein assay, and samples were then passed through 10 kDa amicon filters at 14,000 g, 30 min at 4 degrees to remove proteins from the sample. Flow-throughs were separated into reactions for NADH and NAD + . To one of these samples, HCl was added to 10 mM and samples heated at sample heated at 70C for 30 min to degrade NADH, leaving behind NAD + alone. For both aliquots, 25 µL sample was then added to 100 µL ADH cycling mix (0.2 mg/ml alcohol dehydrogenase enzyme, 2% ethanol, 100 mM Tris pH 8.5). Samples were allowed to cycle for 10 min at room temperature, followed by 50 µL addition of an MTT/PMS solution (0.1 mM phenazine methosulfate, 0.8 mM 3-(4,5-Dimethylthiazol-2-yl)-2,5-diphenyltetrazolium bromide), 100 mM Tris–HCl pH 8.5). Plates were then incubated for 15 min and absorbance was measured at 570 nM. NAD concentrations were extrapolated from a standard curve, and normalised to protein concentrations determined by BCA protein assay.

### In vivo* xenograft studies*

All animal protocols described were approved by the IBIS and HUVR Institutional Animal Care and Use Committee (0309-N-15). Xenografts were generated by the subcutaneous injection of 2 × 10^6^ RPMI-2650 or 1 × 10^6^ Detroit-562 cells, tumorspheres, or 1 × 10^4^ positive or negative cells previously sorted by cytometer into the right flanks of 6- to 8-week-old female athymic nude mice. Cells or tumorspheres were suspended 1:1 in Matrigel (Corning) prior to the injection. Animals were examined twice a week, and tumors were measured using calipers. Mice were sacrificed when the experiments were terminated, the tumors reached 300 mm^3^, or the clinical endpoint was reached.

### In vivo* xenograft treatment*

Tumors from the xenografts were extracted and fractionated for reimplantation subcutaneously in nude mice. Two weeks later, mice were randomly assigned to different groups to be treated for three weeks with GNE617 (30 mg/kg in 60%PEG40-10%ethanol-30%D5 W, p.o. 5 days and 5 days off), GMX1778 (200 mg/kg in 60% PEG40-10% ethanol-30% D5 W, p.o. once a week), cisplatin (2 mg/kg in PBS, i.p. twice a week), 2HNA (200 mg/kg in 10%DMSO-40%PEG300-5%Tween80, i.p. four days a week), and docetaxel (15 mg/kg in 5% DMSO-40% PEG300-5% Tween 80–50% H2O, i.p. twice a week). Once the experiment was completed, the tumor measurement continued for two weeks more.

### Flow cytometry/FACS sorting

Cells were suspended in buffer (PBS containing 2% FBS and 5 mM EDTA), blocked with human blocking reagent (Miltenyi Biotec) for 10 min at 4 °C, and incubated with the appropriate antibodies (Miltenyi Biotec): CD10-FITC (130–124-215), CD184-PE (130–117-690), CD19-APC (130–113-642), CD133-PE (130–113-108), CD166-PE (130–118-349) and CD44-APC (130–113-331). After washing the cells twice with buffer, the cells were analyzed by a FACS Canto II cytometer (BD Biosciences) or sorted by a Fusion and Astrios cytometer (BD Biosciences).

### Statistical analysis

Statistical analyses of the experiments were performed using GraphPad Prism. Data were compared with control data using the unpaired Student’s t test or Student’s t test with Welch’s correction. P values less than 0.05 were considered significant and are represented according to the following classification: *p* < 0.05 (*), *p* < 0.01 (**) and *p* < 0.001 (***).

## Supplementary Information

Below is the link to the electronic supplementary material.

**Additional file 1.**

## Data Availability

The datasets used and/or analyzed during the current study are available from the corresponding author on reasonable request.
