# Supplementary material for: NAD pool as an antitumor target against cancer stem cells in head and neck cancer
Source: J Exp Clin Cancer Res. 2023 Mar 3;42:55. doi: 10.1186/s13046-023-02631-2 (PMC9983242; doi:10.1186/s13046-023-02631-2)

**Supplementary Information**

# NAD POOL AS AN ANTITUMOR TARGET AGAINST CANCER STEM CELLS IN HEAD AND NECK CANCER

LE Navas et al

**Supplementary table 1**. Analysis of CD10, CD184, CD19, CD133, CD166 and CD44 positive subpopulations by FACS in HNSCC cell lines.

| **(%±SD)** | **CD10+** | **CD184+** | **CD19+** | **CD133+** | **CD166+** | **CD44+** |
| --- | --- | --- | --- | --- | --- | --- |
| RPMI-2650 | 1.018±0.27 | 0.801±0.73 | 1.721±1.45 | 0.056±0.12 | 60.75±1.48 | 99.68±0.20 |
| Detroit-562 | 0.609±0.41 | 0.185±0.10 | 0.388±0.28 | 0.049±0.05 | 99.63±0.23 | 99.76±0.17 |

**Supplementary Table 2.** Differential genes common to CD10, CD184, CD19 and NAMPT subpopulations obtained from transcriptomic analysis. All the genes in color are related to tumorigenic process, acting as oncogenes (in red), tumor suppressor genes (in blue), ambiguous genes depending on the type of the tumor (in green).

| **NAMPT**  **CD10** | | **NAMPT**  **CD184** | | | **NAMPT**  **CD19** | **NAMPT**  **CD10**  **CD184** | **NAMPT**  **CD10**  **CD184**  **CD19** |
| --- | --- | --- | --- | --- | --- | --- | --- |
| *NFYC*  *EIF3C*  *KIF1B*  *ABCC5*  *SEC31A*  *AP2M1*  *PMS2*  *SLC35F6*  *SH3GLB2*  *MKRN1*  *LARP1B*  *FBXL18*  *ARHGEF10*  *BCS1L*  *TAF1C*  *KMT2E*  *GNB2*  *ERC1* | *PAPSS1*  *MED17*  *TUBD1*  *RALBP1*  *PIGT*  *CERS2*  *SCARA3*  *CNIH1*  *NFKBIA*  *MARK3*  *GTF2IP1*  *DIABLO*  *DAB2*  *TANGO2*  *FKTN*  *HIPK1*  *PRPF31*  *JUP* | *ZNF74*  *VDAC2*  *RHOBTB3*  *YPEL5*  *IGFBP3*  *C9ORF72*  *NARF*  *RDX*  *MSH6*  *RNMT*  *RRBP1*  *CASP8*  *SHMT2*  *JMJD1C*  *REPIN1*  *RXRB*  *F11R*  *CCNL1*  *SARNP*  *DOCK9*  *DDX11* | *GUCD1*  *TOM1L1*  *GATD1*  *CCNB1*  *P4HA1*  *OPA1*  *EIF4G1*  *SLC11A2*  *NAA80*  *PHETA1*  *FUT8*  *MBNL1*  *GIT2*  *NR3C1*  *DERL1*  *SMIM7*  *GNAI1*  *MON2*  *EPS15L1*  *SCAF11*  *STX3* | *PTK2*  *TXNRD2*  *RASGRP1*  *NDUFV1*  *TP53I11*  *PEX13*  *SIN3B*  *RAB5IF*  *CNOT6L*  *IL1R1*  *CCDC90B*  *STX1A*  *NEDD1*  *LINC02210*  *MYH10*  *TLCD3A*  *WASH6P*  *LIN9*  *PDXDC1*  *AURKB*  *MICOS10* | *C1QBP*  *DGKZ*  *PRMT5*  *FKBP10*  *CD63*  *DDB1*  *PUM1*  *STAU1*  *ZNF189*  *RPL29*  *MACROH2A1*  *ARHGAP21*  *MATR3*  *SIRT1*  *ELOVL1*  *RIOK3*  *RO60*  *TM2D1* | *MELK*  *PKM*  *AKT2*  *ATP5F1B*  *MORF4L2*  *SAMHD1*  *GOSR2*  *EHMT2*  *UACA*  *PRMT2*  *KAT6B*  *ABCA5* | *DDX3X*  *NFE2L1*  *GLUL* |
| N=36 | | N=63 | | | N=18 | N=13 | N=3 |

**Supplementary Table 3**: IC50s for NAMPT inhibitors in parental and NAMPT CRISPR clones of both cell lines.

|  | **IC50 (nM)** | |
| --- | --- | --- |
| **Cell line** | **GNE617** | **GMX1778** |
| **RPMI-2650** | 2.76 | 1.84 |
| **R46** | 0.18 | 0.10 |
| **R48** | 0.28 | 0.27 |
| **Detroit-562** | 5.19 | 5.68 |
| **D11** | 1.20 | 1.12 |
| **D16** | 1.55 | 1.84 |


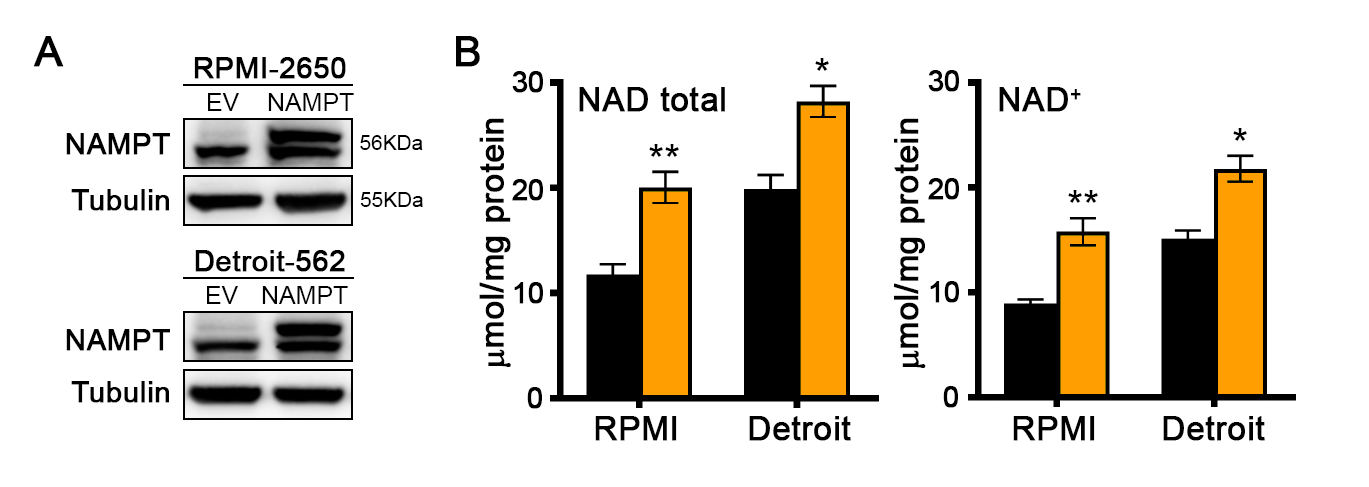


**Supplementary figure 1:** Verification of NAMPT overexpression in RPMI and Detroit HNSCC cell lines. A: Western blot showing increased NAMPT ectopic overexpression. B: increased NAD total and NAD+ pools in cells overexpressing NAMPT (in orange).


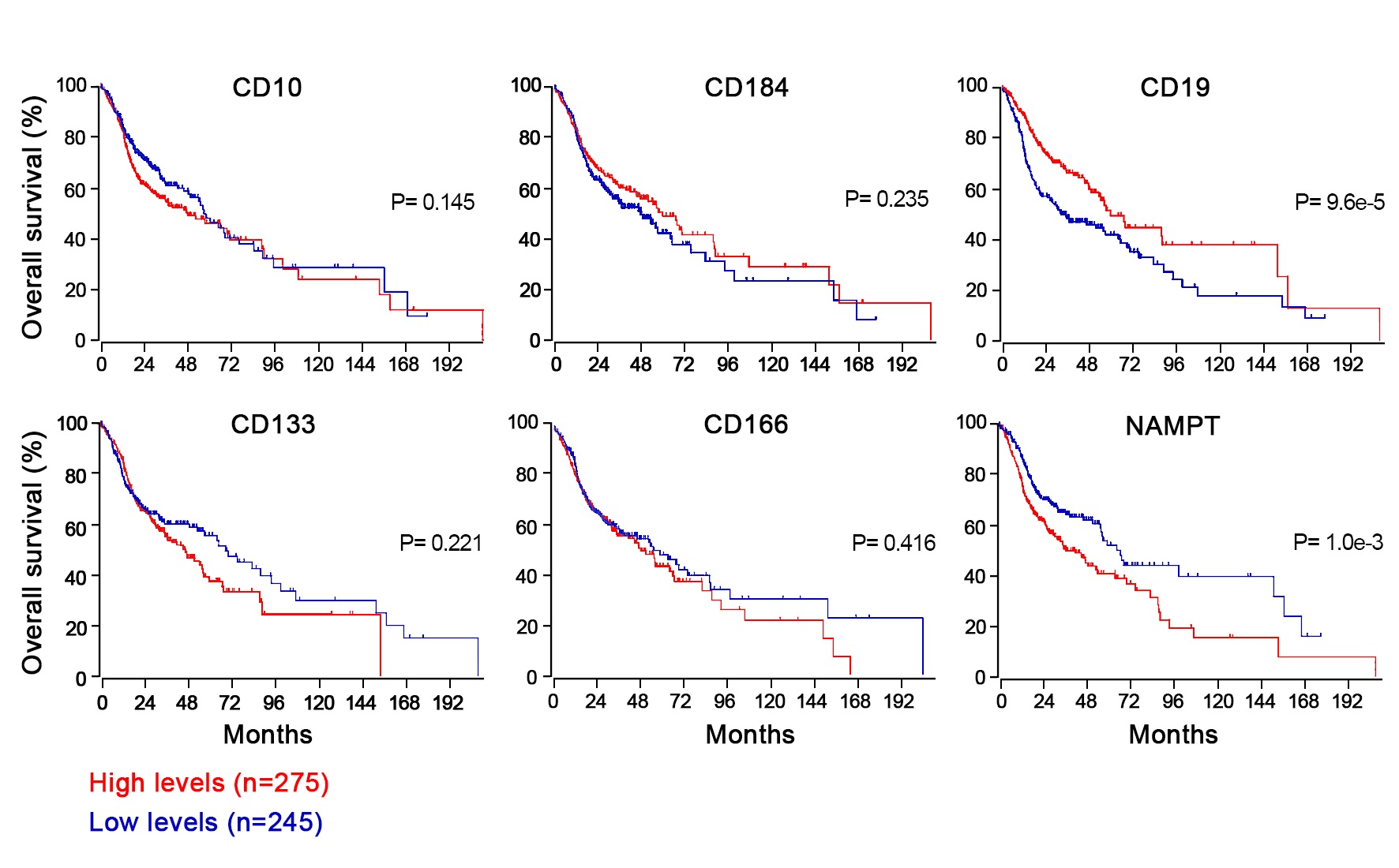


**Supplementary Figure 2. Overall survival of HNSCC patients from the TCGA database.** Kaplan-Meier curves show the overall survival of HNSCC patients with high and low expression levels of *CD10*, *CD184*, *CD19*, *CD133*, *CD166* and *NAMPT* genes from the TCGA (The Cancer Genome Atlas) database, N=520.


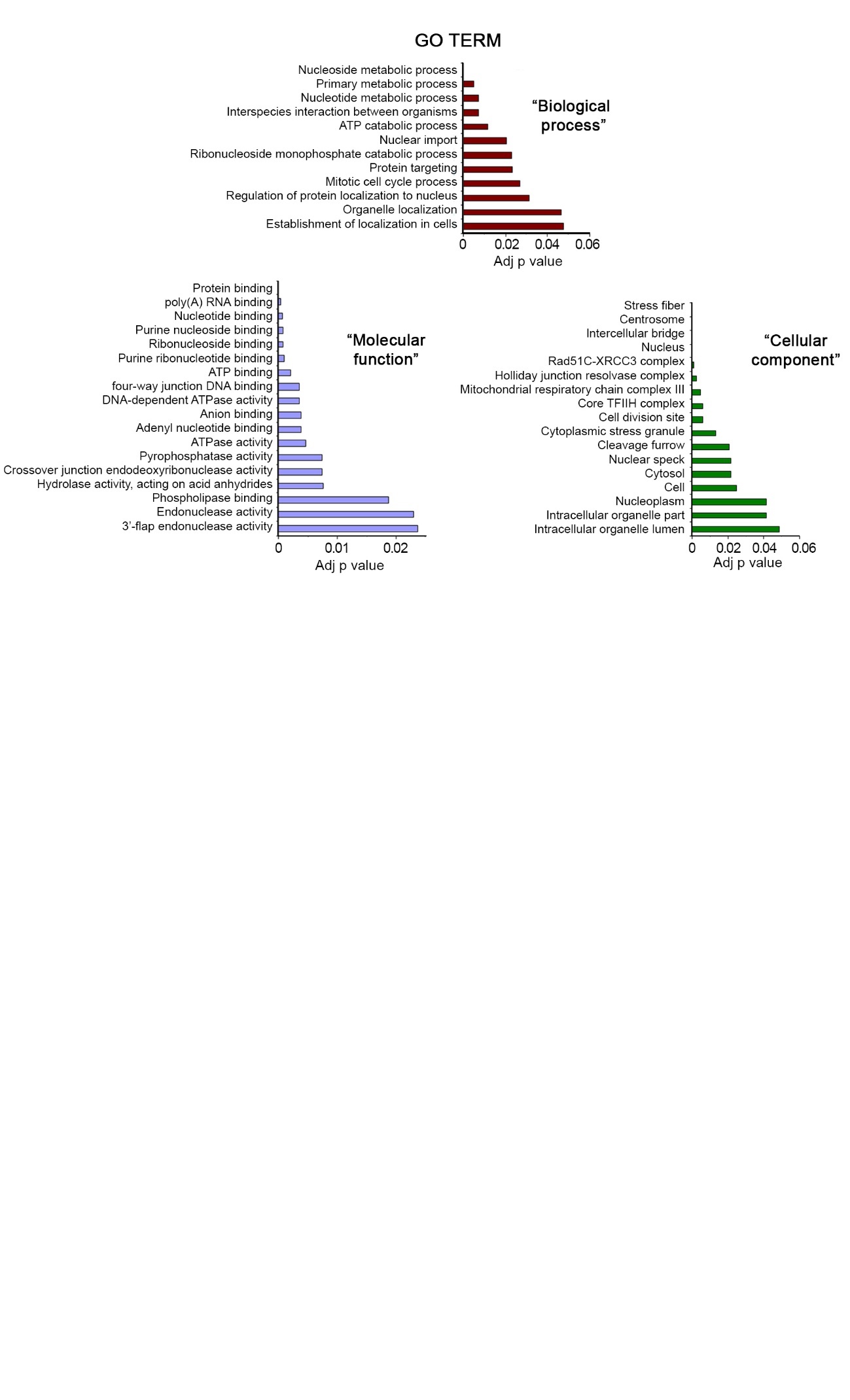


**Supplementary Figure 3. GO term analysis.** Analysis of the genes by the terms GO biological process, molecular function and cellular component (p<0.05).


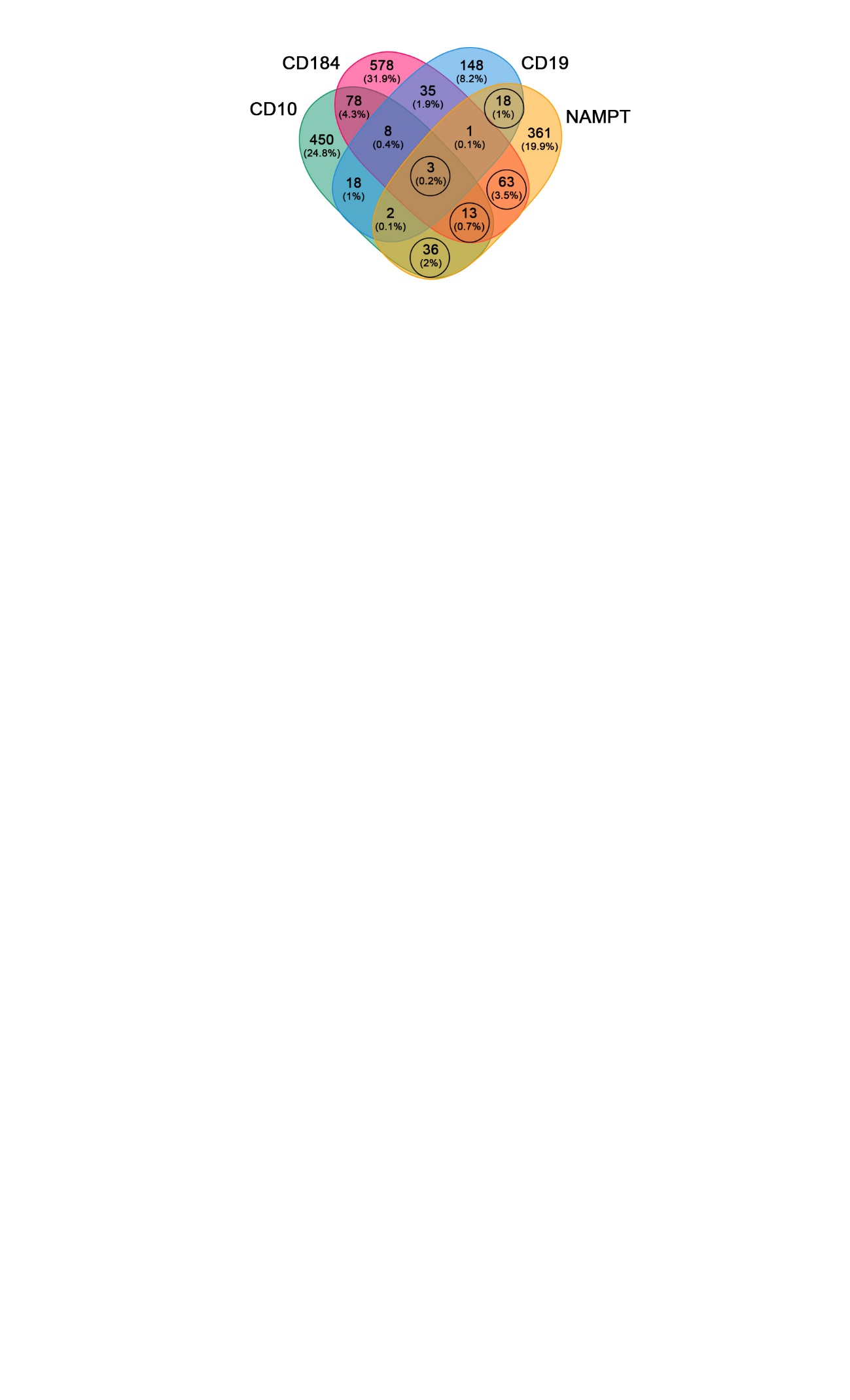


**Supplementary Figure 4. Transcriptomic analysis of differential genes common to CD10, CD184, CD19 and NAMPT subpopulations in HNSCC cell lines**. Venn diagram represents the differential genes common to positive and negative CD10, CD184 and CD19 populations and NAMPT overexpression and CRISPRs in RPMI-2650 and Detroit-562 cell lines obtained by NGS sequencing.


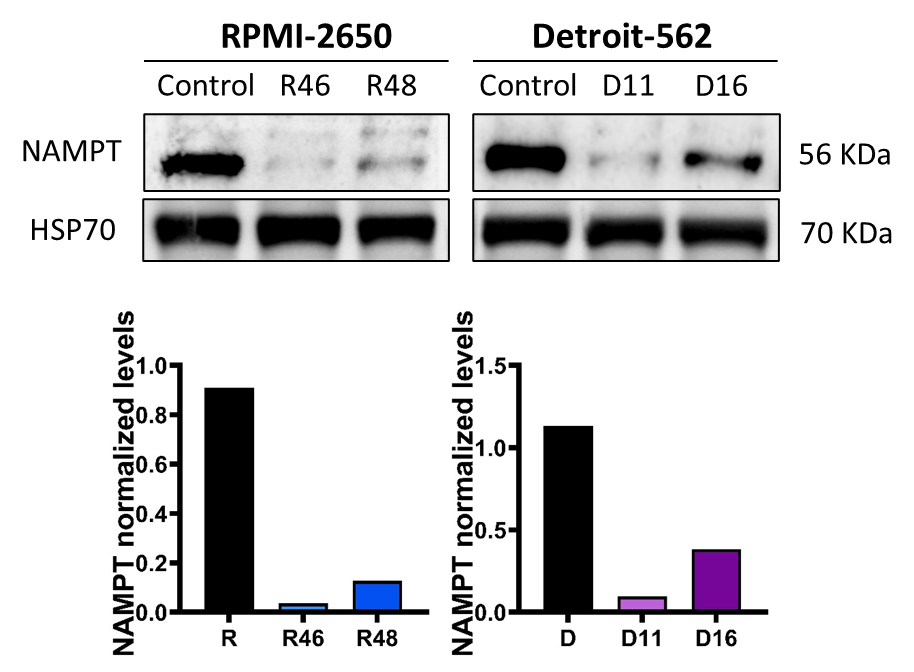


**Supplementary Figure 5**: Densitometric quantification of NAMPT expression in the WB of figure 2C.


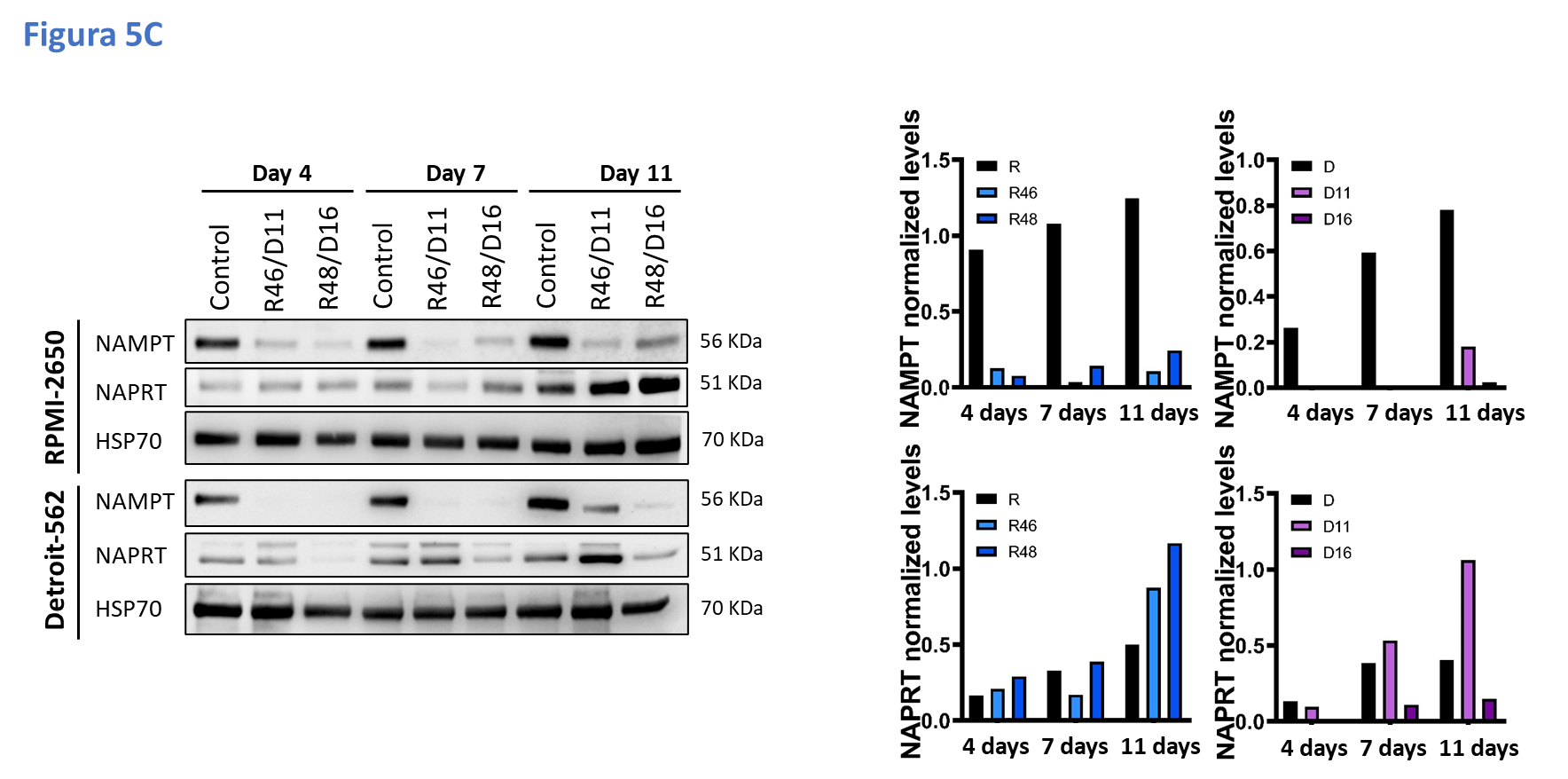


**Supplementary Figure 6**: Densitometric quantification of NAMPT and NAPRT expression in the WB of Figure 5C.

**Supplementary materials**

**Original images of western blots**


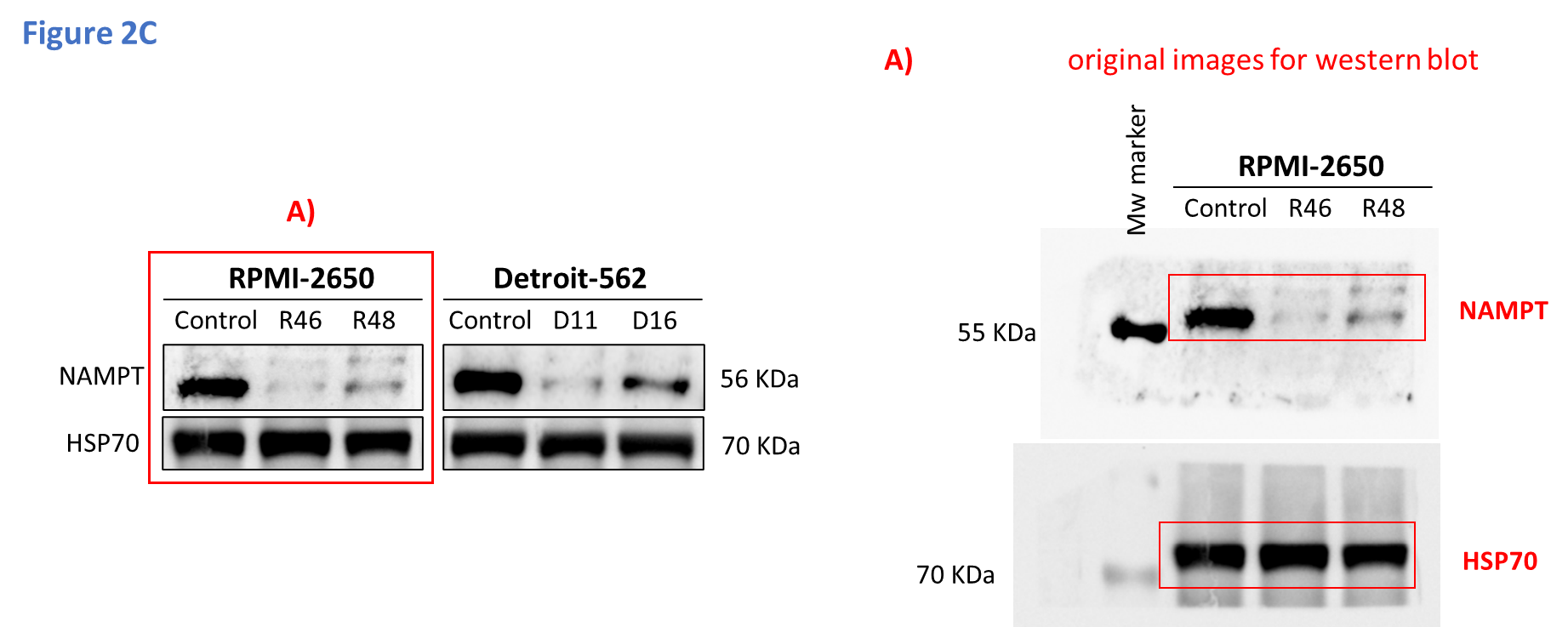


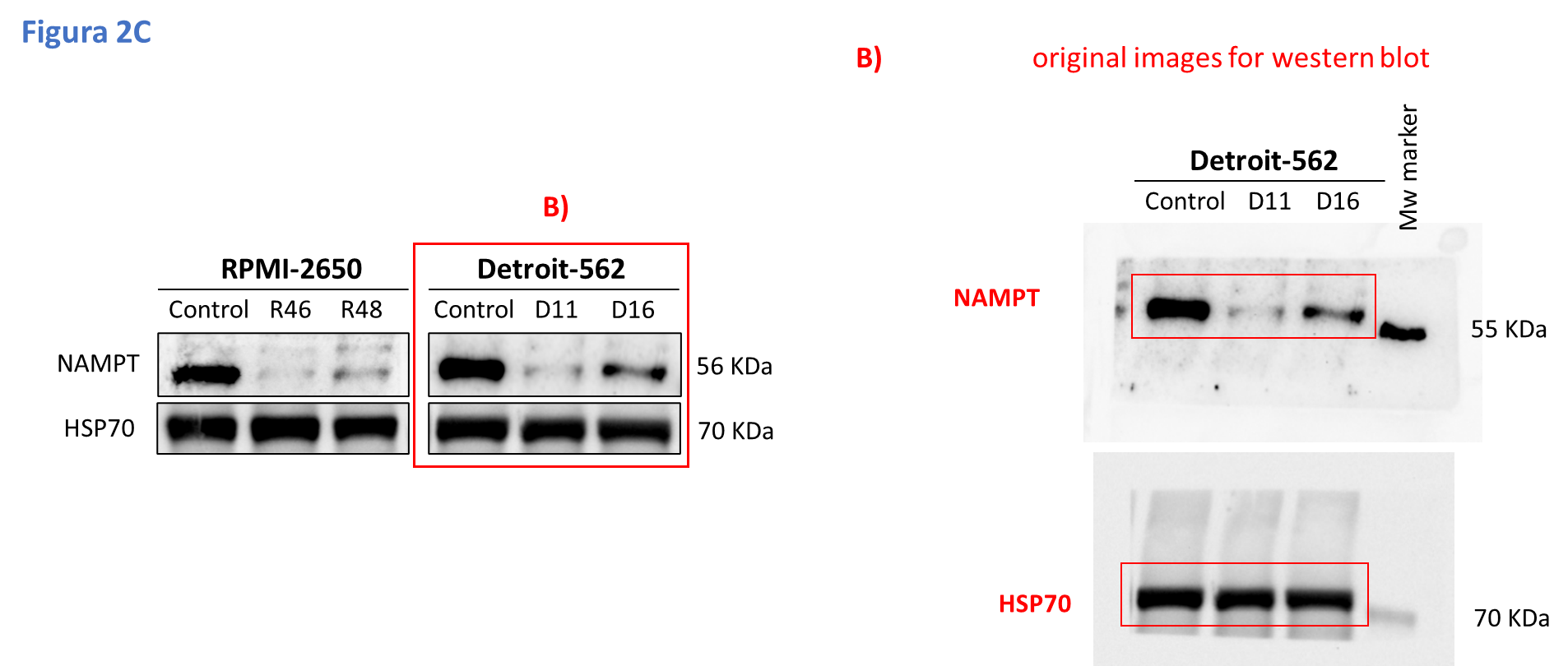


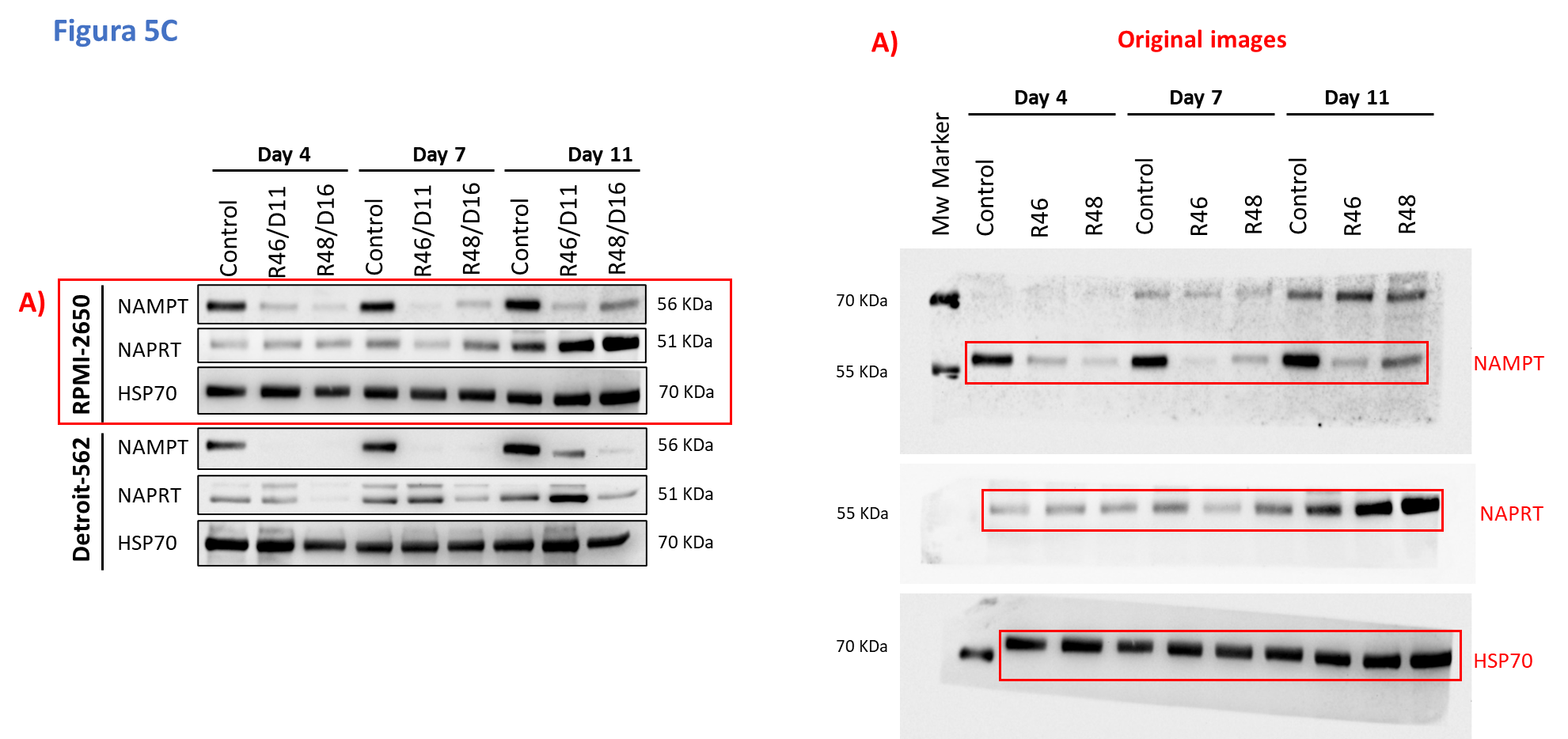


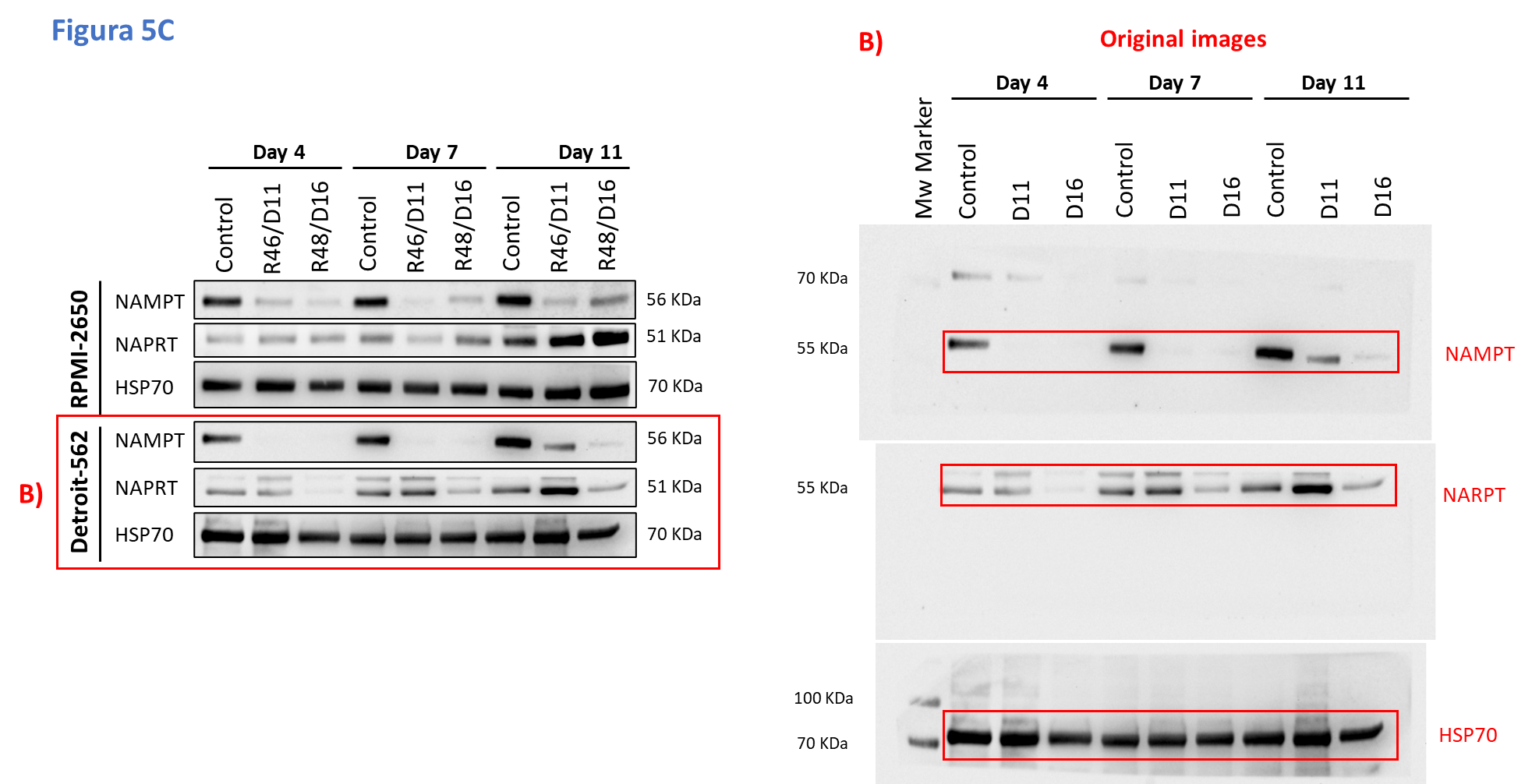

Supplement: Supplementary file 1 — Additional file 1. [file 13046_2023_2631_MOESM1_ESM.doc]
